# Electrical lymph node scanning (ELS) system for real-time intra-operative detection of involved axillary lymph nodes in adjuvant breast cancer patients

**DOI:** 10.1038/s41598-024-61600-7

**Published:** 2024-06-05

**Authors:** Fereshteh Abbasvandi, Reihane Mahdavi, Mahdis Bayat, Farzane Hajighasemi, Fahimeh Jahanbakhshi, Faeze Aghaei, Nafiseh Sami, Batoul Khoundabi, Hossein Ataee, Narges Yousefpour, Parisa Hoseinpour, Seyed Mohamad Sadegh Mousavi Kiasary, Maryam Omrani Hashemi, Fatemeh Shojaeian, Atieh Akbari, Najmeh Bagherhosseini, Afshin Moradi, Mohammad Esmaeil Akbari, Mohammad Abdolahad

**Affiliations:** 1 ATMP Department, Breast Cancer Research Center, Motamed Cancer Institute, ACECR, P.O. Box 1517964311, Tehran, Iran; 2 Nano Bioelectronics Devices Lab, Cancer Electronics Research Group, School of Electrical and Computer Engineering, Faculty of Engineering, University of Tehran, Tehran, Iran; 3 Cancer Research Center, Shahid Beheshti University of Medical Sciences, Tehran, Iran; 4 Student Research Committee, Faculty of Medicine, Tehran Medical Sciences, Islamic Azad University, Tehran, Iran; 5 Iran-Helal Institute of Applied Science and Technology, Red Crescent Society of Iran, Tehran, Iran; 6 Research Center for Health Management in Mass Gathering, Red Crescent Society of the Islamic Republic of Iran, Tehran, Iran; 7 SEPAS Pathology Laboratory, Tehran, Iran; 8 Department of Pathology, Shohada Hospital, Shahid Beheshti University of Medical Sciences, Tehran, Iran; 9 Cancer Institute, Imam-Khomeini Hospital, Tehran University of Medical Sciences, Tehran, Iran

**Keywords:** Breast cancer surgery, Nodal staging, Pathology, Electrical impedance spectroscopy (EIS), Impedance phase slope (IPS), Cancer, Engineering

## Abstract

Lymph node (LN) status is an essential prognostic factor in breast cancer (BC) patients, with an important role in the surgical and therapeutic plan. Recently, we have been developed a novel system for real-time intra-operative electrical LN scanning in BC patients. The ELS scores were calibrated by pathological evaluation of the LNs. Herein, we evaluated the efficacy of ELS in a prospective study for non-chemo-treated breast cancer patients. This is a prospective study in which ELS scores are blind for pathologists who declare the clearance or involvement of LNs based on permanent pathology as the gold standard. ELS and frozen-section (FS) pathology results were achieved intra-operatively, and samples were sent for the permanent pathology. The score of ELS did not affect the surgeons’ decision, and the treatment approach was carried out based on FS pathology and pre-surgical data, such as imaging and probable biopsies. Patients were recruited from October 2021 through November 2022, and 381 lymph nodes of 97 patients were included in the study. In this study we recruited 38 patients (39.2%) with sentinel lymph node biopsy (SLNB) and 59 patients (60.8%) with ALND. Of the 381 LNs scored by ELS, 329 sentinel LNs underwent routine pathology, while others (n = 52) underwent both FS and permanent pathology. ELS showed a sensitivity of 91.4% for node-positive patients, decreasing to 84.8% when considering all LNs. Using ROC analysis, ELS diagnosis showed a significant AUC of 0.878 in relation to the permanent pathology gold standard. Comparison of ELS diagnosis for different tumor types and LN sizes demonstrated no significant differences, while increasing LN size correlated with enhanced ELS sensitivity. This study confirmed ELS’s efficacy in real-time lymph node detection among non-chemo-treated breast cancer patients. The use of ELS’s pathological scoring for intra-operative LN diagnosis, especially in the absence of FS pathology or for non-sentinel LN involvement, could improve prognosis and reduce complications by minimizing unnecessary dissection.

## Introduction

Despite the advances in early diagnosis and therapeutic approaches of breast cancer (BC), as one of the most prevalent malignant solid tumors, this is still the second causes of cancer death among women worldwide^1–4^. Apart from various classifications of breast tumors due to the difference in the natural history and aggression of cancer cells, lymph node (LN) involvement can be a predictor of tumor’s behavior. In previous studies, the adverse prognostic effect of LNs metastasis has been widely established^5–8^. In light of this matter, accurate detection of LNs involvement can have a major influence on treatment plans, including the surgical procedures of BC patients^6^. To address this concern, various conventional pre- and intra-operative methods including Ultrasound (US) imaging, fine needle aspiration (FNA), core needle biopsy (CNB), and frozen-section (FS) of sentinel lymph node have been applied for BC patients^9–12^.

Despite past therapeutic approaches to perform complete axillary lymph node dissection (ALND) or lymphadenectomy for BC patients, several types of recent research demonstrated that ALND offers minimal or no oncological benefit in overall survival^13–15^. On the other hand, lymphadenectomy could be associated with detrimental complications, including a reduction in the protective role of LNs for the immune system, lymphedema, seroma formation, limitations of shoulder mobility, and loss of sensitivity^16–19^. Therefore, over the past 30 years, the evolution of axillary surgery in BC treatment has been leaning towards de-escalating invasive interventions, leading to minimizing surgical morbidity and ensuring optimum patient quality of life, while maintaining oncological safety^20^. Recently, the practice has transitioned from routine ALND towards sentinel lymph node biopsy (SLNB), which has become the standard intra-operative method for nodal staging and even towards the complete avoidance of axillary surgery for certain patients. The complication rate of SLNB is significantly lower with 5% compared to 58.4% for ALND^19^. Although SLNB is a less invasive procedure, still up to 50% of the collected SLNBs are involved with tumoral cells and require radical axillary surgery^21,22^.

Considering this ongoing endeavor for accurate identifying of LNs status and decreasing the invasiveness of axillary staging, predictive tools play an important role and several novel techniques have been developed, including spectroscopic or tomographic approaches, such as Raman^23,24^, Electrical impedance (in median frequencies)^25–27^, and Terahertz spectroscopies^28^. Despite the numerous studies documenting the applications of electrical impedance spectroscopy (EIS) in various cancer-related areas like tumor detection, lymph node (LN) involvement, and lymphedema diagnosis^27,29–36^, the pursuit of creating efficient and cost-effective pre-operative or intra-operative diagnoses for metastatic LNs remains hampered by resource-intensive processes and non-clinical approval constraints^37^. Developing such innovations can substantially contribute towards fine-tuning and individualizing patient care strategies, guiding the approach of BC treatment towards less invasiveness without compromising the outcomes.

This clinical research trial is designed on a recently developed system named electrical lymph node spectroscopy (ELS)^38^, focused on detecting LN status during BC surgery of adjuvant patients. This method is based on an EIS system that analyses two electrical impedance features of the nodes named Z_1kHz_ and impedance phase slope (IPS). Given that not all centers possess FS facilities, this diagnostic tool could serve as a valuable aid in addressing this issue. Furthermore, it has the potential to detect non-sentinel lymph node involvement, a task not typically undertaken in routine pathology procedures.

## Materials and methods

### Patients recruitment and methodology

This research was conducted at a referral breast cancer center in Iran (Breast Cancer Institute of Shohada-e-Tajarish Hospital) from October 2021 through November 2022. Ninety-seven non-chemo-treated breast cancer patients that were candidates for LN dissection (SLNB or ALND) were recruited in the study. All 381 dissected LNs were intra-operatively scored by ELS and evaluated by permanent pathology as the gold standard^38^. The ELS were also compared with FS pathology results. The institutional review board of Tehran University of Medical Science (IR.TUMS.VCR. REC.1397.355) approved the test protocol and all methods were performed in accordance with the relevant guidelines and regulations. All the patients provided written informed consent.

**Figure 1:**
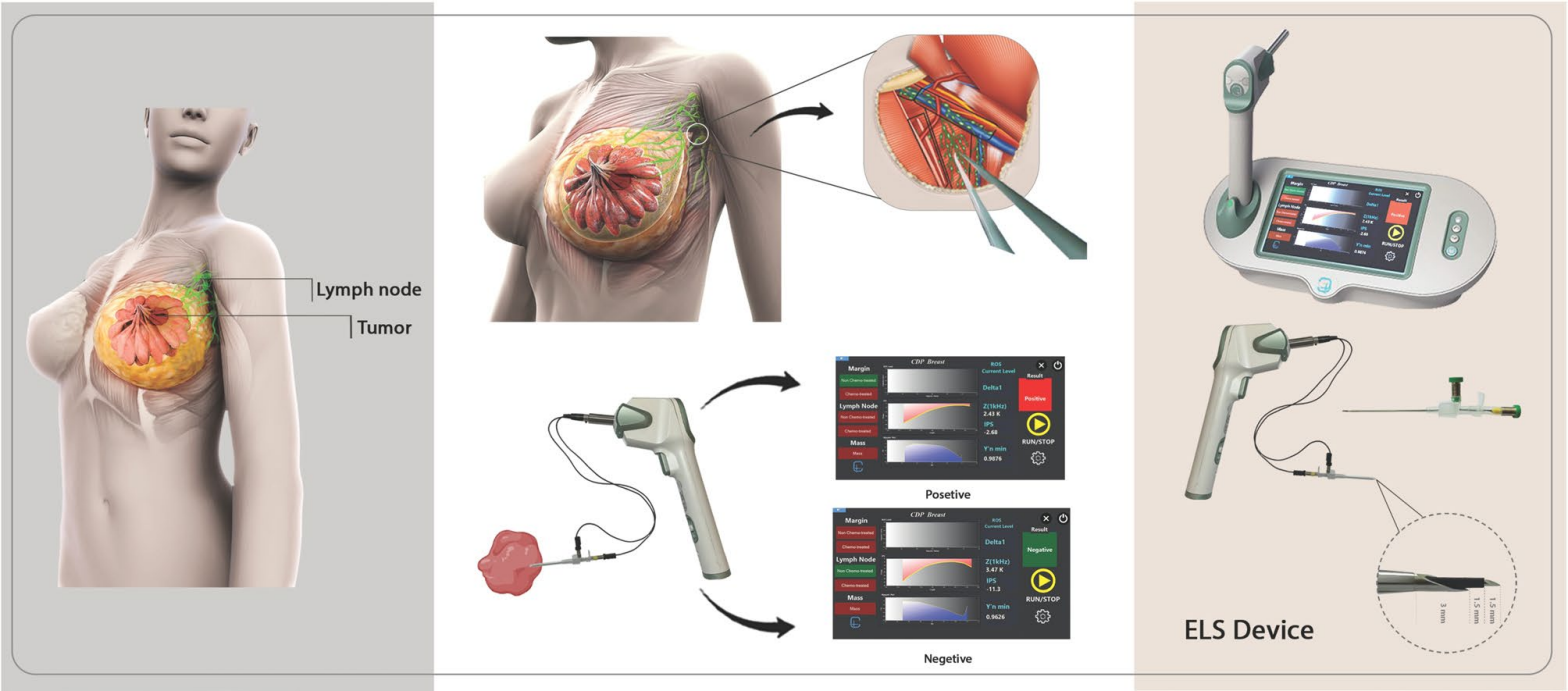
A schematic of ELS device.

To evaluate the reliability of ELS scoring in clinical situations, we applied ELS for detecting LN involvement in both SLNB and ALND surgical plans (i.e., for both sentinel and non-sentinel LNs), during breast conserving surgery (BCS) or mastectomy (Fig. 1).

The ELS probe was used on at least three to six suspicious regions of each dissected LN, including the cortex and inner parts. Conventionally, we first entered the needle in more adenotic and stiffer areas. The impedance measurement, feature extraction, and response analysis stages took only about 5 s for each measurement, so each lymph node diagnosis by ELS took about 1 min in total. All the tested LNs were then labeled and sent for FS or permanent pathology according to the surgeons’ plan through pre- and intra-operative observations. The ELS response is based on analyzing the dielectric properties of LN samples obtained via two impedimetric classification parameters of Z_1kHz_ and IPS. The ELS responses were then compared with permanent pathology results as the gold standard. The H&E and IHC assays of LNs tested by ELS were investigated blindly by two independent and experienced pathologists, and a final consensus was established in the case of inconsistency.

### ELS structure and measurement protocols

ELS is a needle-based electrical impedance-based measurement system designed for intra-operative detection of LN involvement. LNs at least 5 mm in diameter are evaluated with two medical-grade stainless steel needles. This is a precise system that calculates and analyses impedance magnitude and phase diagrams of the tested media in a two-electrode configuration. Two impedance-based features identifying intracellular, extracellular, and cell membrane abnormalities can accurately discriminate normal and involved LNs. The LN involvement diagnosis may occur through two mechanisms: first, detecting fat content consumption and reduction due to the shifting the metabolism pathway from hypoxia glycolysis in primary tumor site to fatty acid oxidation (FAO) in the LN environment, leads to decrease the electrical impedance modulus, and second the increment of membrane permeability of the cancerous epithelial cells that entered the LN structure in high frequencies. In conclusion, ELS detects involved LNs by detecting cancerous cells existence in the LN environment (direct), and unusual consumption of fat content of the LN media (indirect). Some experimental data released in Fig. 3 of our pervious manuscript, convience us to choose the two parameters of impedance magnitude in the frequency of 1 kHz (Z_1kHz_) and impedance phase slope in the frequency ranges of 100–500 kHz (IPS), which are the best fitting experimental cutoffs for discrimination of involved LNs from clear ones in adjuvant patients^38^. These two parameters of impedance magnitude, Z_1kHz_ and impedance phase slope (IPS) are extracted, and the final diagnosis is made according to the previous classification criteria for discrimination of normal and involved LNs.

### Statistical analysis

In this manuscript, SPSS software (version 24) was utilized for the statistical analysis of the study. Ordinal variables were presented using number and percentage reports. Moreover, quantitative variables were reported through utilizing mean/median. According to the type of variables, charts statistics were used. The mean/median of two groups were compared by T-TEST or Mann–Whitney test. Kolmogorov–Smirnov test was used for checking the normality of quantitative variables. Also, the correlation of ordinal variables was done using one of the two Chi-square tests or Fisher’s exact test. To assess the diagnostic accuracy of electrical impedance spectroscopy methods in comparison with the gold standard (permanent pathology), the ROC analysis was applied. Some other crucial parameters including selectivity, sensitivity, specificity, and accuracy of the EIS scoring, were also evaluated.

## Results

**Figure 2:**
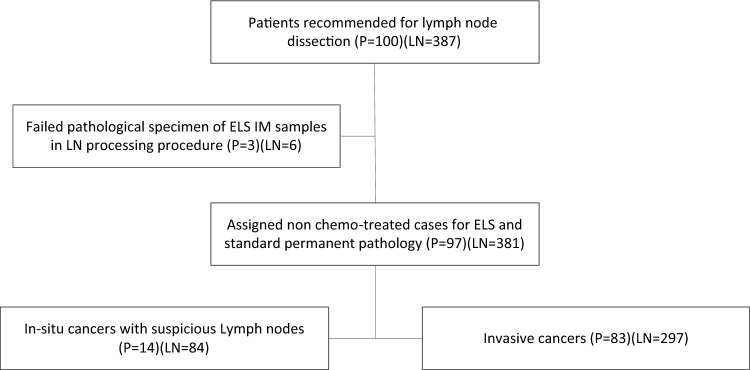
Flowchart of patients.

Initially, 100 patients who were candidate for lymph node dissection in breast cancer surgery were recruited in the study (387 total LNs). Six LNs from three patients were excluded due to pathological assessment failures. Therefore, the cohort study included 381 LNs dissected from 97 patients, of which 297 LNs belong to 83 patients with invasive cancers and 84 other LNs were from 14 patients with carcinoma in situ (Fig. 2).

The clinical information from 97 recruited patients with 381 LNs was evaluated and Table 1 summarized the demographic characteristics and outcome evaluation results of the patients. Table 1 presents the patient's age, tumor grade, tumor size, and cancer type, which were assessed through both pathology and radiology.

**Table 1:** Basic information of patients included in the study.

Variable	Category	n (%)/Mean [Range]
Age		48.23 [25–74]
Cancer type		
CNB	IDC	77 (79.4%)
	ILC	1 (1.0%)
	DCIS	5 (5.2%)
	Other	14 (14.4%)
Permanent pathology	IDC	67 (69.1%)
	ILC	5 (5.2%)
	DCIS	6 (6.2%)
	Other	19 (19.6%)
Tumor grade		
CNB	I	17 (20.0%)
	II	47 (55.3%)
	III	20 (23.5%)
	V	1 (1.2%)
Permanent pathology	I	17 (18.1%)
	II	46 (48.9%)
	III	31 (33.0%)
Radiological diagnosis	suspicious	24 (25.0%)
	Reactive	28 (29.2%)
	Free	44 (45.8%)
Nodal status	N0	50 (57.5%)
	N1	19 (21.8%)
	N2	15 (17.2%)
	N3	3 (3.4%)
Tumor BIRADS (Radiology)	4	50 (56.1%)
	5	34 (38.2%)
	6	5 (5.6%)
Radiological tumor size	(0–2) cm	39 (40.2%)
	(2–5) cm	54 (55.7%)
	> 5cm	4 (4.1%)
Permanent pathology T-staging	T1	17 (19.5%)
	T2	54 (62.1%)
	T3	9 (10.3%)
	T4	1 (1.1%)
	T_is_	6 (6.9%)
Surgical technique	SLNB	38 (39.2%)
	ALND	59 (60.8%)
Estimated LN size (mm)	Total	11.0 [1–50]

*ALND* axillary lymph node dissection, *SLNB* sentinel lymph node biopsy, *IDC* invasive ductal carcinoma.

Different types of breast cancer tumors diagnosed in permanent pathology included invasive ductal carcinoma (IDC) (n = 67), invasive lobular carcinoma (ILC) (n = 5), ductal carcinoma in situ (DCIS) (n = 6), and others (n = 19). The patients’ mean age was 48.23 years old with the range of 25–74.

Table 2 is presenting the sensitivity, specificity, accuracy, positive, and negative predictive values of ELS as an independent real-time LN scoring system in the presence of permanent pathology as the gold standard. It is also reporting mutual comparisons between other techniques. Surgical LN management for n = 38 (39.2%) patients was SLNB, while ALND was performed for n = 59 (60.8%). Of note, among 381 scored LNs by ELS, n = 52 sentinel LNs underwent FS, and others (n = 329) non-sentinel LNs only underwent routine permanent pathology. The total number of positive dissected LNs (confirmed by permanent pathology) was n = 100, while n = 281 LNs were diagnosed as free from tumor cells. First, the results are reported based on the effect of intra-operative ELS diagnosis per patient. Following the results in Table 2, among 33 node-positive patients, the total sensitivity of ELS was about 91.4%, in which n = 32 patients were diagnosed node positive. Only n = 1 patients were miss diagnosed as negative nodes. When we considered each LN as a sample under the test, the total sensitivity decreased to 84.8%. Frozen also could be appropriate method in detection of metastatic LNs by 100.0% sensitivity and 93.5% specificity, in 52 LNs. However, in comparison between radiology and permanent pathology, the results reported just 59.3% sensitivity. *P* values number less than 0.05 identified that those techniques in method columns had acceptable power in reporting nodal status by standard methods.

**Table 2:** Sensitivity, specificity, accuracy, and positive and negative predictive values of all applied methods by time.

Time^$^	Investigation	Method	Standard method	Sensitivity (%)	Specificity (%)	Accuracy (%)	Positive predictive value (%)	Negative predictive value (%)	*P* value
Before surgery	Total patients	Radiology	PP	59.3	47.8	51.0	30.8	75.0	0.729
	Total patients	Radiology	FNA/CNB	76.9	40.0	66.7	76.9	40.0	0.583
	Total patients	FNA/CNB	PP	100.0	41.7	61.1	46.2	100.0	0.902
During surgery	Total patients	Frozen	PP	100.0	93.5	95.2	84.6	100.0	< 0.001*
	Total^α^ (per patient)	ELS	PP	91.4	77.4	82.5	69.6	94.1	< 0.001*
	SLNB^α^	ELS	PP	75.0	89.5	85.2	83.3	86.7	< 0.001*
	ALND^α^	ELS	PP	96.3	72.1	81.4	71.4	100.0	< 0.001*
	Total^β^ (per individual LN)	ELS	PP	84.8	90.8	89.2	76.4	94.5	< 0.001*
	SLNB^β^	ELS	PP	76.9	84.6	82.7	66.7	90.0	< 0.001*
	ALND^β^	ELS	PP	86.0	91.8	90.3	72.0	95.7	< 0.001*

*PP* permanent pathology.

*Significant at 0.05 levels by chi-square or exact fisher tests.

^$^The time is considered for method column.

^α^Results per patients.

^β^Results per individual LN.

**Figure 3:**
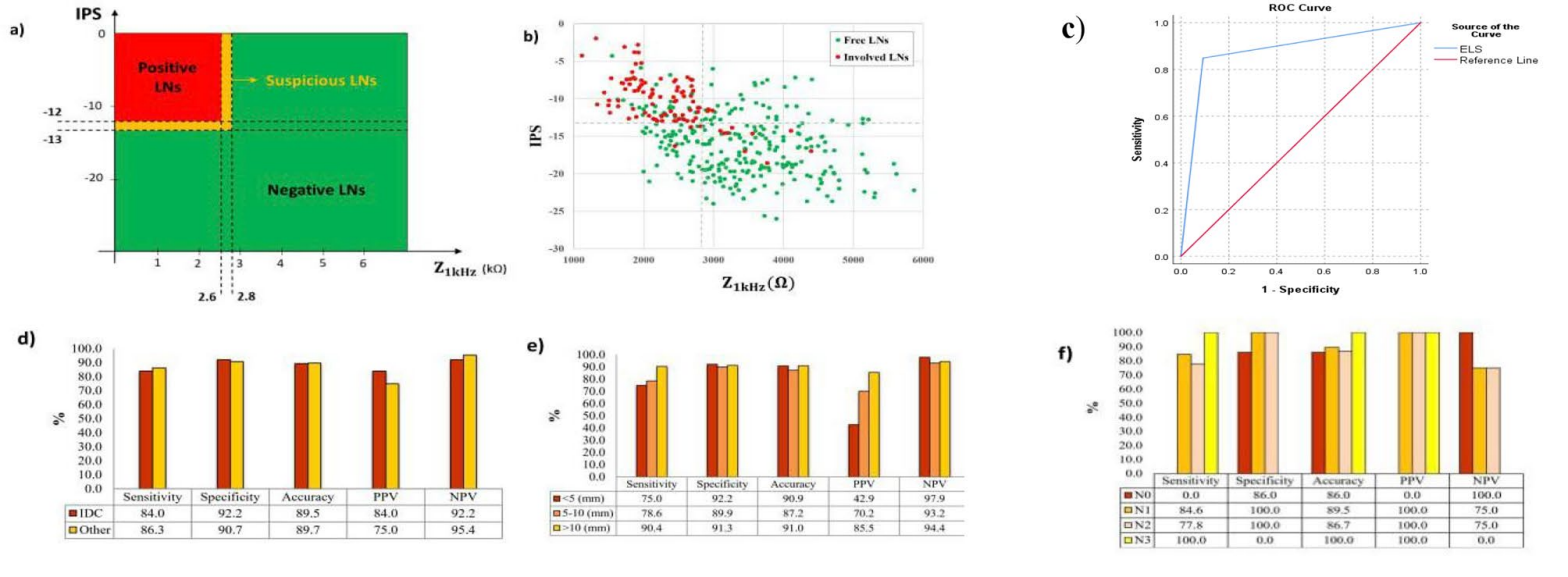
(**a**) Classification criteria of ELS for non-chemo-treated patients discussed in^38^. (**b**) IPS-Z_1kHz_ diagram of ELS in 381 LNs of non-chemo-treated patients according to their pathological evaluations showing complete discrimination between free and involved LNs. (**c**) AUC and ROC for ELS in LNs detection. The reference line is belonged to the permanent pathology as the gold standard. (**d**) Sensitivity, Specificity, Accuracy, Positive, and Negative Predictive Values of ELS results by cancer types. (**e**) Sensitivity, Specificity, Accuracy, Positive, and Negative Predictive Values of ELS results by estimated LN size categories. (**f**) Sensitivity, Specificity, Accuracy, Positive, and Negative Predictive Values of ELS results by TNM (As sample size in N3 type is 3, the comparison was done between three other groups).

For each dissected LN, two classification parameters (Z_1kHz_, IPS) were measured, and according to the classification criteria discussed in^38^ (Fig. 3a), the final ELS score was recorded. The mean values of Z_1kHz_ and IPS were 3.58 kΩ and − 16.8 for pathologically negative (free) LNs, while it was 2.3 kΩ and − 10.1 respectively for positive (involved) LNs, which were similar to previously reported calibration^38^. The recorded classification parameters were projected into a 2-dimensional graph (Z_1kHz_ on X-axis and IPS on Y-axis) for all free (green dots) and involved (red dots) LNs according to Fig. 3b.

To investigate the relationship between ELS diagnosis and permanent pathology gold standard, the ROC analysis was performed (Fig. 3c). As can be seen, significant AUC belonged to ELS diagnosis (AUC = 0.878).

A comparison between ELS diagnosis of IDC and other tumor types has been performed, which revealed no significant difference (Fig. 3d). The comparison among three LN size categories showed a significant difference between them in positive predictive values (*P* < 0.05) (Fig. 3e). Although ELS sensitivity increased with LN size (from 75% in small LNs to 90% in LNs larger than 10 mm), patients' nodal status (N0, N1, N2) did not have any relation with it (Fig. 3f).

The comparison of the mean of three quantitative variables including age, size of tumor and LN using the Mann–Whitney test was done as follows: there was a statistically significant relationship between age and ELS result, the average age of people with a negative result in ELS, was almost 2.5 years older than the average age of people with a positive result in the ELS test, 48.9 ± 9.3 versus 46.5 ± 11.6 (*P* = 0.041). Also, there was a statistically significant relationship between the tumor size and the ELS result. Tumor size of patients with positive ELS was almost 2 units more than patients with negative ELS, (28.7 ± 12.1 vs. 22.7 ± 11.7, *P* = 0.017). The result of comparing the average size of LN between ELS-positive and negative patients (12.6 ± 6.8 and 10.3 ± 7.8 mm, respectively) showed that the average size of LN in patients with positive result was significantly higher than patients with negative ELS (*P* < 0.001). Also, the correlation coefficient test confirmed the aforementioned results by significant Spearman differences between ELS with age, tumor size and LN size (Table 3). Another variable which had significant association with ELS, was lymph vascular invasion (LVI) in CNB with correlation coefficient equal to 0.274 (*P* = 0.022). Regarding tumor grade, the mean difference between tumor grade and ELS in positive and negative ELS results was not significant (*P* = 0.394), as well as the correlation coefficient (*P* = 0.397).

**Table 3:** Spearman's correlation matrix of selected variables.

Variables	Age	Tumor size	LN size	Tumor grade	LVI in CNB
Tumor size	− 0.175 (0.086)				
LN size	− 0.114 (0.266)	0.176 (0.084)			
Tumor grade	− 0.073 (0.487)	0.067 (0.523)	0.077 (0.464)		
LVI in CNB	− 0.229 (0.058)	− 0.006 (0.963)	0.128 (0.296)	0.197 (0.104)	
ELS	− 0.208 (0.041*)	0.244 (0.016*)	0.198 (< 0.001*)	0.088 (0.397)	0.274 (0.022*)

*Significant Spearman correlation between items at 0.05.

(): contains *P* values.

Logistic regression model was performed to investigate the effect of probable risk factors on the odd of a positive permanent pathology result (Table 4). In univariate (unadjusted) model, analysis revealed that patients' age, tumor size, LN size, and ELS result were significant predictors of the permanent pathology of LNs (*P* < 0.05). As the age increased, the odd of positive permanent pathology reduced; with an increase of 1 year in age, the odds ratio was 0.941 (*P* < 0.001). Regarding the size of the tumor, the odd of positive permanent pathology response increased by 1.041 times for every 1 mm increase (*P* = 0.036). High values of LN size given more odds for the permanent test to be positive. Thus, with an increase of 1 mm in the size of LN, the odds ratio for positive result of permanent test, became 1.041 (*P* = 0.007). Notably, the positive results of ELS increased the odd of the permanent test being positive, by 55.138 times (*P* < 0.001). Furthermore, a 1° increase in tumor grade corresponded to an odds ratio of 1.744 for a positive response (*P* = 0.099). In the multiple (adjusted) model, only age and ELS result were significant. The adjusted OR of having a positive result for a LN in the permanent pathology was 84.5-fold higher in patients with ELS positive scores. Moreover, the adjusted OR for patients with a positive score based on patients’ age was 0.909. Examining the LVI in CNB variable to enter the logistic regression model was not confirmed due to the rejection of the Hosmer–Lemshow test, but the association between the results of permanent pathology and LVI in CNB was not significant (*P* = 0.144) based on Fisher’s exact test.

**Table 4:** Univariate and multivariate logistic regression analysis.

Variables	Univariate		Multivariable	
	OR (95% CI)	*P* value	OR (95% CI)	*P* value
Age	0.941 (0.91, 0.96)	< 0.001	0.909 (0.83, 0.98)	0.023
Tumor size	1.041 (1.00, 1.08)	0.036	1.04 (0.97, 1.1)	0.222
Tumor grade	1.744 (0.90, 3.37)	0.099	2.76 (0.97, 7.84)	0.057
LN size	1.041 (1.01, 1.07)	0.007	0.97 (0.88, 1.08)	0.644
ELS	55.13 (27.88, 109.01)	< 0.001	84.5 (14.58, 490.1)	< 0.001

## Discussion

Axillary nodal status is one of the crucial predictor factors for the overall survival of breast cancer patients^39^. Consequently, a great desire is to thoroughly assess the status of LNs during the surgery. As previously mentioned, the therapeutic benefit of extensive surgical dissection of LNs has remained controversial^27^. A very recent systematic review has been proved that ALND’s disadvantages, such as pain, lymphedema, reduction of strength, and range of motion are higher in comparison with sentinel lymph node biopsy and previous estimations^40^. Furthermore, extensive axillary LNs dissection can significantly reduce the immunotherapy efficacy due to the attribution of this therapeutic approach to initiation of systemic immune responses within LNs, especially sentinel lymph nodes^41^. Taking all the vast complications of ALND together, there is an urgent need for accurate detection of tumoral cells in LNs, particularly in patients receiving immunotherapy^41–44^ or even radiotherapy^43^.

From past decades, axillary US imaging is generally a pre-operative non-invasive technique for evaluating nodal status through various ultrasound characteristics of breast tumors such as internal echo^45^. To the best of our knowledge, ultrasonography has been used in a broad range in clinic, while literatures report moderate sensitivity (53.6%) but quite specificity (75.5%) for this method in detection of involved LNs^46^. In light with this not re-assuring reports, our assessments indicated 59.3% sensitivity and 47.8% specificity for US based on permanent pathology as the gold standard.

Currently, FS is one of the most frequently used methods for identifying involved sentinel LNs during BC surgery owing to the high concordance between FS and permanent pathology^47^. In a recent retrospective study, the sensitivity and accuracy of FS was reported 91.89% and 96.73%, in the evaluation of 2230 LNs, respectively^47^. While our study's findings align with previous research in terms of favorable sensitivity, it's essential to acknowledge the drawbacks of FS pathology. These encompass challenges in accurately detecting micrometastasis and tumors with lobular histopathology, as well as factors such as cost, time consumption, and the necessity for an experienced pathologist^47,48^. Regarding the costs, the price of each head probe of ELS system is about 3 dollars, which could be used for each patient. We mean that each head probe can be used more than 10 times in one patient, but in another patient, we must use from another head probe. So, the head probe or the needle probe is disposable for one patient not for one lesion. On the other hand, the price of our system is about 2500 dollars which in comparison with the price of FS system and frozen preparation procedure, it seems that the ELS system could pass the health technology assessment (HTA) criteria^49^. In addition, researches have reported that in about 60–70% of the patients, only sentinel nodes are involved with cancerous cells^13^, so selective completion ALND by applying an accurate intra-operative instrument which could detect metastatic LN, is required. As another novel method, Yuan et al. introduced a modified axillary reverse mapping (ARM) approach called iDEntification and Preservation of ARm lymphaTic system (DEPART), which allows a thorough detection of the axillary lymphatic system and reduction in the arm lymphedema rate without undesirable affecting the morbidity of regional recurrence in BC patients who undergo ALND^50^. Since, the anatomical crossover variations between breast and arm lymph nodes may enhance the risk of metastasis, this concern restricts the widespread usage of the technique by surgeons. Hence, over the past decades, researchers have made great efforts to develop novel and cost-effective techniques for intra-operative identification of metastatic lymph nodes, which could be easily applied to a clinical setting and overcome the morbidities and concerns. Here, the diagnosis of cancer involving LNs with electrical impedance spectroscopy based on an integrated needle electrode called ELS system in a total of 381 LNs (based on the permanent pathology as the gold standard) was carried out successfully, and our data lend credence to the ability of calibrated ELS to detect LN involvement in 97 non-chemo-treated patients.

Taking the various tumor histology types into consideration, lobular histotype has been already characterized as one of the pitfalls of various methods of detecting involved LNs like FNA, FS, and touch preparation (TP) due to the dyshesive nature of the tumor cells. Hence, clinicians should be mindful of the increased likelihood of hidden LN involvement with ILC, potentially leading to early metastasis^47,51–54^. Within our cohort study, we assessed seven excised lymph nodes from the singular patient with invasive lobular carcinoma (ILC) and LN involvement using ELS. This approach yielded an accuracy rate of 85% (6 out of 7). To the best of our knowledge, ELS response is based on analyzing the dielectric properties of LNs, thus it can be expected that the accuration rate of detecting LNs with lobular carcinoma involvement will enhance by utilizing this method. However, reported results could be owing to the low proportion of ILC tumors in our recruited patients, and further evaluation is necessary for an accurate assessment of this matter.

To dive more deeply into the importance of nodal staging, several clinical researches have found numerous breast tumor characteristics associated with LN involvement, such as LVI in CNB and primary tumor size^8,55–57^. Consistent with the previous studies, newly in 2023, Ersoy et al. observed that the presence of LVI had an association with LN involvement in both neoadjuvant and non-neoadjuvant cases^39^. While our investigation unveiled a significant impact of lymphovascular invasion (LVI) in CNB on ELS results, no correlation was observed between this factor and lymph node involvement in permanent pathology. By considering tumor size as another independent prognostic factor of lymph node metastasis, they found that in neoadjuvant cases, tumors greater than 10 mm had higher rate of LN involvement, while in non-neoadjuvant cases none of the patients with pathologic tumor size of 10 mm or smaller had lymph node metastasis^39^. In line with a series of studies, our results illustrated that tumor size is a significant predictor of the permanent pathology of LNs and ELS results (*P* < 0.05). Besides according to our data, the LN sizes can enhance the ELS sensitivity from 75% in small LNs to 90% in LNs larger than 10 mm. These findings underscore the potential of ELS as an innovative histopathologically-calibrated technique, offering valuable assistance in intra-operative decision-making for lymph node identification. Moreover, ELS holds promise for successful navigation through medical trials and gaining consensus approval within clinical committees.

The obtained results confirm the 84.8% sensitivity of ELS in diagnosing LNs, which is more valuable when we focus on the benefits of utilizing such a system in surgery. Therefore, the ELS probe can be useful and widely applied to assist the surgeon during axillary LN surgery. For instance, if a positive sentinel lymph node is diagnosed by FS pathology (or ELS in the absence of FS), and the surgeon goes through performing ALND, all non-sentinel LNs can be checked one by one with the ELS probe. In cases of a positive ELS result, the surgeon should proceed with the removal of subsequent lymph nodes. Conversely, if the ELS probe diagnoses two or more lymph nodes as negative, the surgeon can halt further excisions, effectively managing the number of nodes to be removed while minimizing potential adverse effects. Moreover, this system has the advantage of real‐time scoring, easy usage, and system portability to scan LNs making hopes to inside-the-body detection methods.

Wang et al. raised some concerns about possible artifacts that may hinder ELS's accurate identification of metastatic LNs as an impedance-based detection system^58^. To address this matter, it should be considered that ELS electrodes are directly inserted into dissected LNs and are in close contact with the internal LN structure and cells leading to well-established site binding (in contrast to other devices applied via the patient’s skin). So, some overdiagnoses originating from interfering muscles, vessels, and superficial skin lesions, or underdiagnoses arising from a poor electrical connection (due to high amount of electrode-tissue impedance), high body fat content, air bubbles, and interfering bones have been omitted in this application.

## Conclusion

Precise detection devices and methods are of increased research interest, exemplified by the evaluation of ELS efficacy, an innovative intra-operative lymph node scoring system, within our present study. The results demonstrated about 85% sensitivity and 91% specificity. This system can be utilized in the absence of FS pathology for evaluating sentinel LNs and for evaluating non-sentinel LNs which are not usually conventionally assessed by any pathological method intra-operatively, thus leading to decrease re-operation rate. Moreover, this device has the potential to minimize unnecessary lymph node dissections during surgery, ensuring surgeons in targeting involved nodes effectively. For entering a system as a new clinical tool, we must conduct some clinical trials to achieve to some favorable data to begin health technology assessment in comparison between detection of LNs involvement by ELS with FS during the surgery, and achieving to the standards in a country, for example Iran. So, our next steps will be introducing and designing a clinical trial for achieving HTA of the system in a wide variety of regions in Iran, and after that we can discuss about the probable entrance of the ELS system as a new clinical tool all over the world.

## Data Availability

The datasets used and/or analysed during the current study available from the corresponding author on reasonable request.

## References

[1] Li N, Deng Y, Zhou L, Tian T, Yang S, Wu Y (2019). Global burden of breast cancer and attributable risk factors in 195 countries and territories, from 1990 to 2017: Results from the Global Burden of Disease Study 2017. J. Hematol. Oncol..

[2] Miripour ZS, Ghahremani A, Karimi K, Jahanbakhsh F, Abbasvandi F, Hoseinpour P (2023). Electrochemical therapy (EChT) of cancer tumor with an external anode, a way to achieve pathological complete response. Med. Oncol..

[3] Abdolahad M, Kaviani A (2023). Real-time detection of cellular metabolism: A new trend for intra-operative diagnosis of cavity margins: Intraoperative CDP. Arch. Breast Cancer.

[4] Saadati F, Jahanbakhshi F, Mahdikia H, Abbasvandi F, Ghomi H, Yazdani N (2023). Cold physical plasma toxicity in breast and oral squamous carcinoma in vitro and in patient-derived cancer tissue ex vivo. Appl. Sci..

[5] Dubsky P, Pinker K, Cardoso F, Montagna G, Ritter M, Denkert C (2021). Breast conservation and axillary management after primary systemic therapy in patients with early-stage breast cancer: The Lucerne toolbox. Lancet Oncol..

[6] Hotton J, Salleron J, Henrot P, Buhler J, Leufflen L, Rauch P (2020). Pre-operative axillary ultrasound with fine-needle aspiration cytology performance and predictive factors of false negatives in axillary lymph node involvement in early breast cancer. Breast Cancer Res. Treat..

[7] Fujii T, Yajima R, Tatsuki H, Suto T, Morita H, Tsutsumi S (2015). Significance of lymphatic invasion combined with size of primary tumor for predicting sentinel lymph node metastasis in patients with breast cancer. Anticancer Res..

[8] Sandoughdaran S, Malekzadeh M, Akbari ME (2018). Frequency and predictors of axillary lymph node metastases in Iranian women with early breast cancer. Asian Pac. J. Cancer Prev. APJCP.

[9] Ayana G, Dese K, Choe S-W (2021). Transfer learning in breast cancer diagnoses via ultrasound imaging. Cancers.

[10] Moschetta M, Telegrafo M, Carluccio D, Jablonska J, Rella L, Serio G (2014). Comparison between fine needle aspiration cytology (FNAC) and core needle biopsy (CNB) in the diagnosis of breast lesions. Il Giornale Chir..

[11] Rana MK, Rana APS, Sharma U, Barwal TS, Jain A (2022). Evolution of frozen section in carcinoma breast: Systematic review. Int. J. Breast Cancer.

[12] Reimer T (2023). Omission of axillary sentinel lymph node biopsy in early invasive breast cancer. Breast.

[13] Gatzemeier W, Mann GB (2013). Which sentinel lymph-node (SLN) positive breast cancer patient needs an axillary lymph-node dissection (ALND)–ACOSOG Z0011 results and beyond. Breast.

[14] Javadi S, Akbari ME, Hashemi S, Moradian F, Akbari A, Mohamadi F (2021). Less axillary lymphadenectomy is more beneficial: 27-year Follow-up of patients with breast cancer. Int. J. Cancer Manag..

[15] Zheng Q, Luo H, Xia W, Lu Q, Jiang K, Hong R (2022). Long-term survival after sentinel lymph node biopsy or axillary lymph node dissection in pN0 breast cancer patients: a population-based study. Breast Cancer Res. Treat..

[16] Houssami N, Diepstraten SC, Cody HS, Turner RM, Sever AR (2014). Clinical utility of ultrasound-needle biopsy for preoperative staging of the axilla in invasive breast cancer. Anticancer Res..

[17] Yoshihara E, Smeets A, Laenen A, Reynders A, Soens J, Van Ongeval C (2013). Predictors of axillary lymph node metastases in early breast cancer and their applicability in clinical practice. Breast.

[18] McLaughlin SA, Wright MJ, Morris KT, Giron GL, Sampson MR, Brockway JP (2008). Prevalence of lymphedema in women with breast cancer 5 years after sentinel lymph node biopsy or axillary dissection: Objective measurements. J. Clin. Oncol..

[19] Žatecký J, Coufal O, Holánek M, Kubala O, Kepičová M, Gatěk J (2023). Level I axillary dissection in patients with breast cancer and tumor-involved sentinel lymph node after NAC is not sufficient for adequate nodal staging. Turk. J. Surg..

[20] Panellists, C. *Advanced Breast Cancer Sixth International Consensus Conference (ABC6)*.

[21] Purushotham AD, Upponi S, Klevesath MB, Bobrow L, Millar K, Myles JP (2005). Morbidity after sentinel lymph node biopsy in primary breast cancer: Results from a randomized controlled trial. J. Clin. Oncol..

[22] Feggi L, Basaglia E, Corcione S, Querzoli P, Soliani G, Ascanelli S (2001). An original approach in the diagnosis of early breast cancer: Use of the same radiopharmaceutical for both non-palpable lesions and sentinel node localisation. Eur. J. Nucl. Med..

[23] Svoboda RM, Prado G, Mirsky RS, Rigel DS (2019). Assessment of clinician accuracy for diagnosing melanoma on the basis of electrical impedance spectroscopy score plus morphology versus lesion morphology alone. J. Am. Acad. Dermatol..

[24] Vasudeva S, Thapa R (2021). Colloseum to estimate the accuracy of detection of cervical intraepithelial neoplasia using electrical impedance spectroscopy with colposcopy-A one year study. Indian J. Obstet. Gynecol. Res..

[25] Årsvold, A. T., Zeltner, A. S., Cheng, Z., Schwaner, K. L., Jensen, P. T., & Savarimuthu, T. R. (eds.) Lymph node detection using robot assisted electrical impedance scanning and an artificial neural network. In *2021 International Symposium on Medical Robotics (ISMR)* (IEEE, 2021).

[26] Mentzel H-J, Malich A, Kentouche K, Freesmeyer M, Böttcher J, Schneider G (2003). Electrical impedance scanning—Application of this new technique for lymph node evaluation in children. Pediatr. Radiol..

[27] Malich A, Boehm T, Facius M, Freesmeyer M, Azhari T, Werner B (2002). Electrical impedance scanning of lymph nodes: Initial clinical and technical findings. Clin. Radiol..

[28] Kashihara T, Zablocki D, Sadoshima J (2019). YAP-dependent metabolic remodeling in local lymph node boosts the function of cancer cells. Biotarget.

[29] Comen EA, Norton L, Massagué J (2011). Breast cancer tumor size, nodal status, and prognosis: Biology trumps anatomy. J. Clin. Oncol..

[30] Beheshti Firoozabadi J, Mahdavi R, Shamsi K, Ataee H, Shafiee A, Ebrahiminik H (2022). Intraoperative assessment of high-risk thyroid nodules based on electrical impedance measurements: A feasibility study. Diagnostics.

[31] Mahdavi R, Hosseinpour P, Abbasvandi F, Mehrvarz S, Yousefpour N, Ataee H (2020). Bioelectrical pathology of the breast; Real-time diagnosis of malignancy by clinically calibrated impedance spectroscopy of freshly dissected tissue. Biosens. Bioelectron..

[32] Mahdavi R, Mehrvarz S, Hoseinpour P, Yousefpour N, Abbasvandi F, Tayebi M (2022). Intraradiological pathology-calibrated electrical impedance spectroscopy in the evaluation of excision-required breast lesions. Med. Phys..

[33] Mamou J, Coron A, Hata M, Machi J, Yanagihara E, Laugier P (2010). Three-dimensional high-frequency characterization of cancerous lymph nodes. Ultrasound Med. Biol..

[34] Hong YT, Yun J, Lee JH, Hong K-H (2021). Smart needle to diagnose metastatic lymph node using electrical impedance spectroscopy. Auris Nasus Larynx.

[35] Zandi A, Sh ZD, Shojaeian F, Mousavi-Kiasary SS, Abbasvandi F, Zandi A (2021). The design and fabrication of nanoengineered platinum needles with laser welded carbon nanotubes (CNTs) for the electrochemical biosensing of cancer lymph nodes. Biomater. Sci..

[36] Uygun ZO, Yeniay L, Sağın FG (2020). CRISPR-dCas9 powered impedimetric biosensor for label-free detection of circulating tumor DNAs. Anal. Chim. Acta.

[37] Lin Y-C, Chen S-C, Chang H-K, Hsueh S, Tsai C-S, Lo Y-F (2005). Identifying good prognosis group of breast cancer patients with 1–3 positive axillary nodes for adjuvant cyclophosphamide, methotrexate and 5-fluorouracil (CMF) chemotherapy. Jpn. J. Clin. Oncol..

[38] Mahdavi R, Yousefpour N, Abbasvandi F, Ataee H, Hoseinpour P, Akbari ME (2021). Intraoperative pathologically-calibrated diagnosis of lymph nodes involved by breast cancer cells based on electrical impedance spectroscopy; A prospective diagnostic human model study. Int. J. Surg..

[39] Ersoy E, Elsayad M, Pandiri M, Knee A, Cao QJ, Crisi GM (2023). Intraoperative lymph node assessment (touch preparation only) for metastatic breast carcinoma in neoadjuvant and non-neoadjuvant settings. Arch. Pathol. Lab. Med..

[40] Bakri NAC, Kwasnicki RM, Khan N, Ghandour O, Lee A, Grant Y (2023). Impact of axillary lymph node dissection and sentinel lymph node biopsy on upper limb morbidity in breast cancer patients: A systematic review and meta-analysis. Ann. Surg..

[41] Rahim MK, Okholm TLH, Jones KB, McCarthy EE, Liu CC, Yee JL (2023). Dynamic CD8+ T cell responses to cancer immunotherapy in human regional lymph nodes are disrupted in metastatic lymph nodes. Cell.

[42] Reticker-Flynn NE, Engleman EG (2023). Lymph nodes: At the intersection of cancer treatment and progression. Trends Cell Biol..

[43] Fransen MF, van Hall T, Ossendorp F (2021). Immune checkpoint therapy: Tumor draining lymph nodes in the spotlights. Int. J. Mol. Sci..

[44] Van Pul KM, Fransen MF, Van de Ven R, De Gruijl TD (2021). Immunotherapy goes local: The central role of lymph nodes in driving tumor infiltration and efficacy. Front. Immunol..

[45] Koehler K, Ohlinger R (2010). Sensitivity and specificity of preoperative ultrasonography for diagnosing nodal metastases in patients with breast cancer. Ultraschall Med. Eur. J. Ultrasound.

[46] Zhang H, Sui X, Zhou S, Hu L, Huang X (2019). Correlation of conventional ultrasound characteristics of breast tumors with axillary lymph node metastasis and Ki-67 expression in patients with breast cancer. J. Ultrasound Med..

[47] Nair NS, Das A, Shet T, Kirti K, Hawaldar R, Desai S (2023). Accuracy of intraoperative frozen section analysis of lymph nodes in women undergoing axillary sampling for treatment of breast cancer: Single institution audit. Clin. Breast Cancer.

[48] Namdar ZM, Omidifar N, Arasteh P, Akrami M, Tahmasebi S, Nobandegani AS (2021). How accurate is frozen section pathology compared to permanent pathology in detecting involved margins and lymph nodes in breast cancer?. World J. Surg. Oncol..

[49] DiNardo LJ, Lin J, Karageorge LS, Powers CN (2000). Accuracy, utility, and cost of frozen section margins in head and neck cancer surgery. Laryngoscope.

[50] Yuan Q, Wu G, Xiao S-Y, Hou J, Ren Y, Wang H (2019). Identification and preservation of arm lymphatic system in axillary dissection for breast cancer to reduce arm lymphedema events: A randomized clinical trial. Ann. Surg. Oncol..

[51] Elliott RM, Shenk RR, Thompson CL, Gilmore HL (2014). Touch preparations for the intraoperative evaluation of sentinel lymph nodes after neoadjuvant therapy have high false-negative rates in patients with breast cancer. Arch. Pathol. Lab. Med..

[52] Guidroz JA, Johnson MT, Scott-Conner CE, De Young BR, Weigel RJ (2010). The use of touch preparation for the evaluation of sentinel lymph nodes in breast cancer. Am. J. Surg..

[53] Ohashi R, Matsubara M, Watarai Y, Yanagihara K, Yamashita K, Tsuchiya S (2016). Diagnostic value of fine needle aspiration and core needle biopsy in special types of breast cancer. Breast Cancer.

[54] Cocco D, ElSherif A, Wright MD, Dempster MS, Kruse ML, Li H (2021). Invasive lobular breast cancer: Data to support surgical decision making. Ann. Surg. Oncol..

[55] Verheuvel N, Ooms H, Tjan-Heijnen V, Roumen R, Voogd A (2016). Predictors for extensive nodal involvement in breast cancer patients with axillary lymph node metastases. Breast.

[56] Malter W, Hellmich M, Badian M, Kirn V, Mallmann P, Kraemer S (2018). Factors predictive of sentinel lymph node involvement in primary breast cancer. Anticancer Res..

[57] Cornwell LB, Mcmasters KM, Chagpar AB (2011). The impact of lymphovascular invasion on lymph node status in patients with breast cancer. Am. Surg..

[58] Wang W, Dong H (2022). A commentary on" Intraoperative pathologically-calibrated diagnosis of lymph nodes involved by breast cancer cells based on electrical impedance spectroscopy; a prospective diagnostic human model study"(Int J Surg 2021; 96: 106166). Int. J. Surg..

